# Non‐lesional white matter in relapsing–remitting multiple sclerosis assessed by multicomponent T2 relaxation

**DOI:** 10.1002/brb3.3334

**Published:** 2023-12-02

**Authors:** Pietro Bontempi, Umberto Rozzanigo, Sabrina Marangoni, Elena Fogazzi, Daniele Ravanelli, Lucia Cazzoletti, Bruno Giometto, Paolo Farace

**Affiliations:** ^1^ Department of Engineering for Innovation Medicine University of Verona Verona Italy; ^2^ Neuro‐radiology Unit, Hospital of Trento Azienda Provinciale per i Servizi Sanitari (APSS) Trento Italy; ^3^ Neurology Unit, Hospital of Trento Azienda Provinciale per i Servizi Sanitari (APSS) Trento Italy; ^4^ Physics department University of Trento Povo Trento Italy; ^5^ Medical Physics Department, Hospital of Trento Azienda Provinciale per i Servizi Sanitari (APSS) Trento Italy; ^6^ Unit of Epidemiology and Medical Statistics, Department of Diagnostics and Public Health University of Verona Verona Italy

**Keywords:** lesion segmentation, multiple sclerosis, Myelin Water Imaging, T2 relaxometry

## Abstract

**Introduction:**

The purpose of the study is to investigate, by T2 relaxation, non‐lesional white matter (WM) in relapsing–remitting (RR) multiple sclerosis (MS).

**Methods:**

Twenty stable RR MS patients underwent 1.5T Magnetic Resonance Imaging (MRI) with 3D Fluid‐Attenuated Inversion‐Recovery (FLAIR), 3D‐T1‐weighted, and T2‐relaxation multi‐echo sequences. The Lesion Segmentation Tool processed FLAIR images to identify focal lesions (FLs), whereas T1 images were segmented to identify WM and FL sub‐volumes with T1 hypo‐intensity. Non‐lesional WM was obtained as the segmented WM, excluding FL volumes. The multi‐echo sequence allowed decomposition into myelin water, intra‐extracellular water, and free water (Fw), which were evaluated on the segmented non‐lesional WM. Correlation analysis was performed between the non‐lesional WM relaxation parameters and Expanded Disability Status Scale (EDSS), disease duration, patient age, and T1 hypo‐intense FL volumes.

**Results:**

The T1 hypo‐intense FL volumes correlated with EDSS. On the non‐lesional WM, the median Fw correlated with EDSS, disease duration, age, and T1 hypo‐intense FL volumes. Bivariate EDSS correlation of FL volumes and WM T2‐relaxation parameters did not improve significance.

**Conclusion:**

T2 relaxation allowed identifying subtle WM alterations, which significantly correlated with EDSS, disease duration, and age but do not seem to be EDSS‐predictors independent from FL sub‐volumes in stable RR patients. Particularly, the increase in the Fw component is suggestive of an uninvestigated prodromal phenomenon in brain degeneration.

## Graphical abstract

In this study, stable relapsing−remitting MS patients were investigated by conventional imaging, WM T2−relaxation, and a fully automated post−processing. The T2−relaxation method allowed the identification of subtle alterations in non lesional WM, particularly an increase of the free water (Fw) component, which correlates with EDSS, disease duration, and patient age. Such increase in the Fw component is suggestive of an unexplored prodromal phenomenon in brain degeneration.

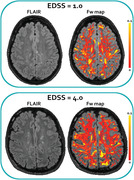

## 1 INTRODUCTION

The association between common neuroradiological markers of multiple sclerosis (MS) and clinical disability is weak, a phenomenon known as the clinical–radiological paradox. There is a limited correlation between white matter (WM) lesion load and functional cognitive impairment (Uher et al., 2017). The correlation between T2 lesion load and the Expanded Disability Status Scale (EDSS) is also moderate, with a stronger correlation with T1 lesion load, consistent with the finding that this subgroup of lesions represents areas of more severe tissue damage (Ciccarelli et al., 2002).

An explanation for this was that rating scales and MRI measure fundamentally different manifestations of MS (Hobart et al., 2004). As this was first raised, there has been considerable progress on several fronts, to a degree that the mismatch between clinical and MRI measures is less an unexplained paradox and more a potentially reconcilable challenge (Chard & Trip, 2017). Attempts to resolve the clinical–radiological paradox should adopt a more multidimensional approach to understanding WM lesions with simultaneous consideration of multiple elements of the quantifiable pathology, using optimum measurement techniques for MRI feature quantification (Mollison et al., 2017).

Of relevance, changes in both normal‐appearing WM and diffusely abnormal WM have been of interest in recent years, as they are independent pathological entities in the disease (West et al., 2014). Non‐lesional abnormalities correlate more strongly with disability than lesion burden and provide new insight into the basis of abnormalities in normal‐appearing WM (Brier et al., 2021). The correlation with EDSS of abnormalities in normal‐appearing WM detected by advanced diffusion techniques (Sowa et al., 2019) indicates that processes outside lesions are important for disability in MS. Quantitative MRI techniques are superior to conventional MRI regarding their sensitivity to subtle alterations within normal‐appearing WM (Granziera et al., 2021). Different techniques and metrics showed normal‐appearing WM damage (Lipp et al., 2019). Among them, myelin water (Mw) imaging (Choi et al., 2019; Lee et al., 2021) was investigated, demonstrating normal‐appearing WM alteration in MS patients compared to healthy controls (Rahmanzadeh et al., 2021). In particular, T2 relaxation can detect tissue damage in the normal‐appearing WM that is missed by conventional imaging (Neema et al., 2009). Furthermore, a nonplaque MRI abnormality that is present in at least 25% of MS patients is diffusely abnormal WM (Lipp et al., 2019), and T2 relaxation was capable of evidencing such microstructural changes in relapsing–remitting (RR) MS (Papadaki et al., 2021).

The present study aims to investigate, in RR MS, the quantitative T2‐relaxation parameters computed on the non‐lesional WM. These quantitative parameters were tested for correlation with the EDSS, disease duration, patient age, and lesion load.

## 2 MATERIALS AND METHODS

### 2.1 Subjects and MR acquisitions

Twenty patients (17 females/3 males, ages 21–69‐year old), affected by RR MS with stable disease course were recruited in an institutional review board‐approved study (internal protocol number: A697). All participants signed written informed consent prior to data collection. Patients’ characteristics are reported in Table 1. All the patients underwent EDSS assessment at MRI and 1 year later without changes (except patient 8 with EDSS from 4.0 to 3.5 and #10 with EDSS from 1.0 to 2.0).

**TABLE 1:** Patients’ characteristics.

**Age (years, median/range)**	38 (20–62)
**Sex (male/female)**	3/17
**Disease duration (years, median/range)**	14 (2–30)
**Subjects under treatment (*n*, %)**	20 (100%)
**Current therapy (*n*)**	Alemtuzumab (1) Fingolimod (3) Fumarate (1) Glatiramer acetate (1) Interferon 2 Natalizumab (9) Teriflunomide 3
**EDSS score at MRI (median/range)**	2 (1–4)

Abbreviations: EDSS, expanded disability status scale.

All the patients underwent MRI examination at 1.5 T, including high‐resolution 3D T1‐weighted and FLAIR sequences and multicomponent T2 relaxation by a multi‐echo gradient and spin echo (GraSE) sequence. Detailed scan parameters are reported in Table 2.

**TABLE 2:** Scan parameters.

	T1‐weighted	FLAIR	Multi‐echo
**Mode**	3D	3D	3D
**Pulse sequence**	gradient‐echo	SE∖IR	GraSE^a^
**Pixel size (mm^2^)**	0.98 × 0.98	0.87 × 0.87	1.64 × 1.64
**Matrix size**	256 × 256	288 × 288	128 × 126^b^
**Slices thickness (mm)**	1.0	1.2	5.0^c^
**Averages**	1	2	1
**Total acquisition time (s)**	222	293	532
**TR (ms)**	9.4	4800	1000
**TE (ms)**	4.6	348	10^d^
**TI (ms)**	–	1600	–
**Flip angle (degrees)**	8	90	90
**Measurements**	WM segmentation	FL segmentation	Mw, IEw, Fw, T2‐IEw

Abbreviations: FL, focal lesion; Fw, free water; GraSE, gradient and spin echo; IEw, intra‐extracellular water; Mw, myelin water; WM, white matter.

^a^ Epi factor = 5.

^b^ Reconstructed at 256 × 256.

^c^ Eighteen slices reconstructed to 36 slices 2.5 mm thickness.

^d^ Thirty‐two equally spaced echoes ranging from 10 to 320 ms.

Multi‐echo images were processed by MATLAB (The MathWorks) and MERA (https://github.com/markdoes/MERA) to perform multi‐exponential relaxation analysis, fitting the data with a distribution of decaying exponential functions and fitting the refocusing flip angle to minimize the impact of stimulated echoes (Prasloski et al., 2012). Multi‐echo data were processed to identify in each voxel (Meyers et al., 2009) three compartments from the obtained T2 spectrum (Bontempi, Rozzanigo, et al., 2021; Bontempi, Scartoni, et al., 2021). The T2 component below 40 ms (MacKay & Laule, 2016) was labeled as Mw, between 40 and 250 ms was labeled as intra/extracellular water (IEw), and above 250 ms was considered cerebrospinal fluid/free water (Fw). Additionally, the T2 map of the IEw compartment (T2‐IEw) was considered in the analysis. The T2 time for the Mw and the Fw compartments was not investigated, given that the shortest and longest sampled echo times were only 10 and 320 ms, respectively, and the estimate would not be accurate (Alonso‐Ortiz et al., 2018; Prasloski et al., 2012).

**FIGURE 1:**
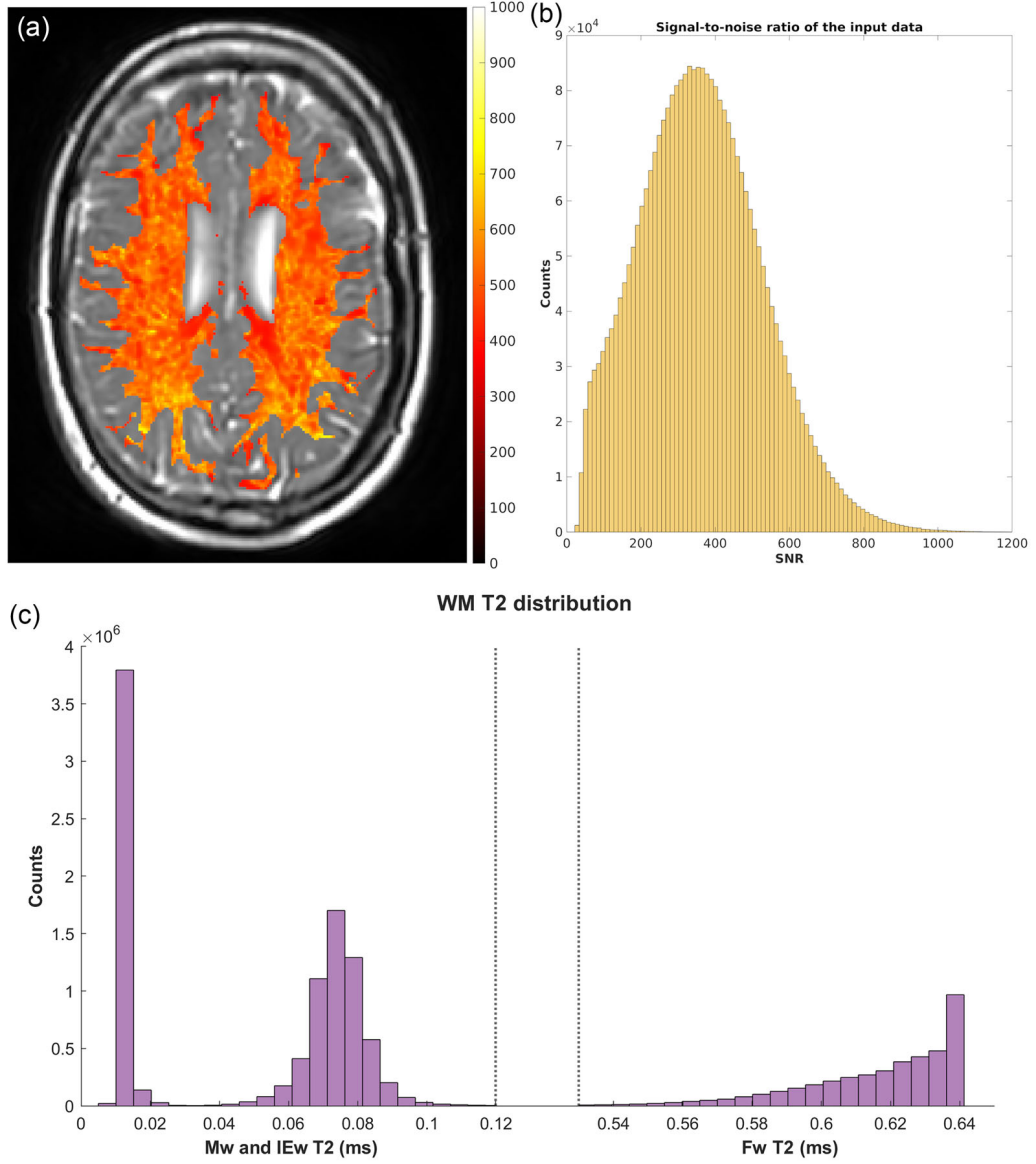
Data quality: (**a**) Example of the SNR map, calculated voxel‐wise as the sum of the spectra divided by the standard deviation of the residual of the fit (this is a standard output of MERA), overlayed on the sixth echo of the gradient and spin echo (GraSE) sequence of patient 6; (**b**) SNR distribution, calculated voxel‐wise as in panel (a) of all the white matter (WM) voxels of all the patients included in the study; (**c**) T2 distribution of all the WM voxels of all the patients included in the study.

Information about comprehensive data quality is reported in Figure 1.

### 2.2 Image segmentation

In each patient, T1 images were processed by FreeSurfer (Reuter et al., 2012). FreeSurfer was utilized to extract brain tissue and to automatically segment WM and distinguish it from non‐WM regions. In particular, T1 hypo‐intense lesions in WM regions are not labeled WM by FreeSurfer.

Image registration was performed by Advanced Normalization Tools (Avants et al., 2011). In detail, FLAIR images were aligned to T1‐weighted images, which were aligned with the corresponding WM mask to GraSE images. All the registrations were performed with an affine transformation, setting 12 degrees of freedom (i.e., rotations, translations, scale, and shear are allowed).

To better estimate WM T2‐relaxation parameters, the focal lesion (FL), segmented as below described, and the peripheral voxels, potentially affected by the partial volume effect, were excluded. To exclude peripheral voxels, the obtained non‐lesional WM mask, registered on GraSE images, was eroded by 1 pixel using functionality available in MATLAB.

FLAIR images were processed by the lesion prediction algorithm of the toolbox (Schmidt et al., 2019) Lesion Segmentation Tool (LST), where a probability threshold equal to 0.5 was used to identify FL. These volumes were further subdivided into sub‐volumes according to the T1 classifications, that is, WM and non‐WM, where non‐WM regions identify T1 hypo‐intensities.

### 2.3 Correlation analysis

Correlation analysis with clinical status assessed by EDSS was performed by measures of the volumes and sub‐volumes of the FL and quantitative T2‐relaxation parameters of the non‐lesional WM.

Quantitative T2 relaxation on the non‐lesional WM was assessed by computing the mean and median of Mw, IEw, Fw, and T2‐IEw, respectively.

For each WM T2‐relaxation parameter, the corresponding *r*‐coefficients and *p*‐values were computed for Spearman's correlation coefficient with EDSS, and for Pearson's correlation coefficient with disease duration, and patient age.

The mutual correlation between FL volumes and T2‐relaxation WM parameters was also assessed by Pearson's correlation coefficient. Finally, the combination of FL volumes and T2‐relaxation WM parameters was assessed as a predictor of EDSS by a bivariate analysis.

## 3 RESULTS

**FIGURE 2:**
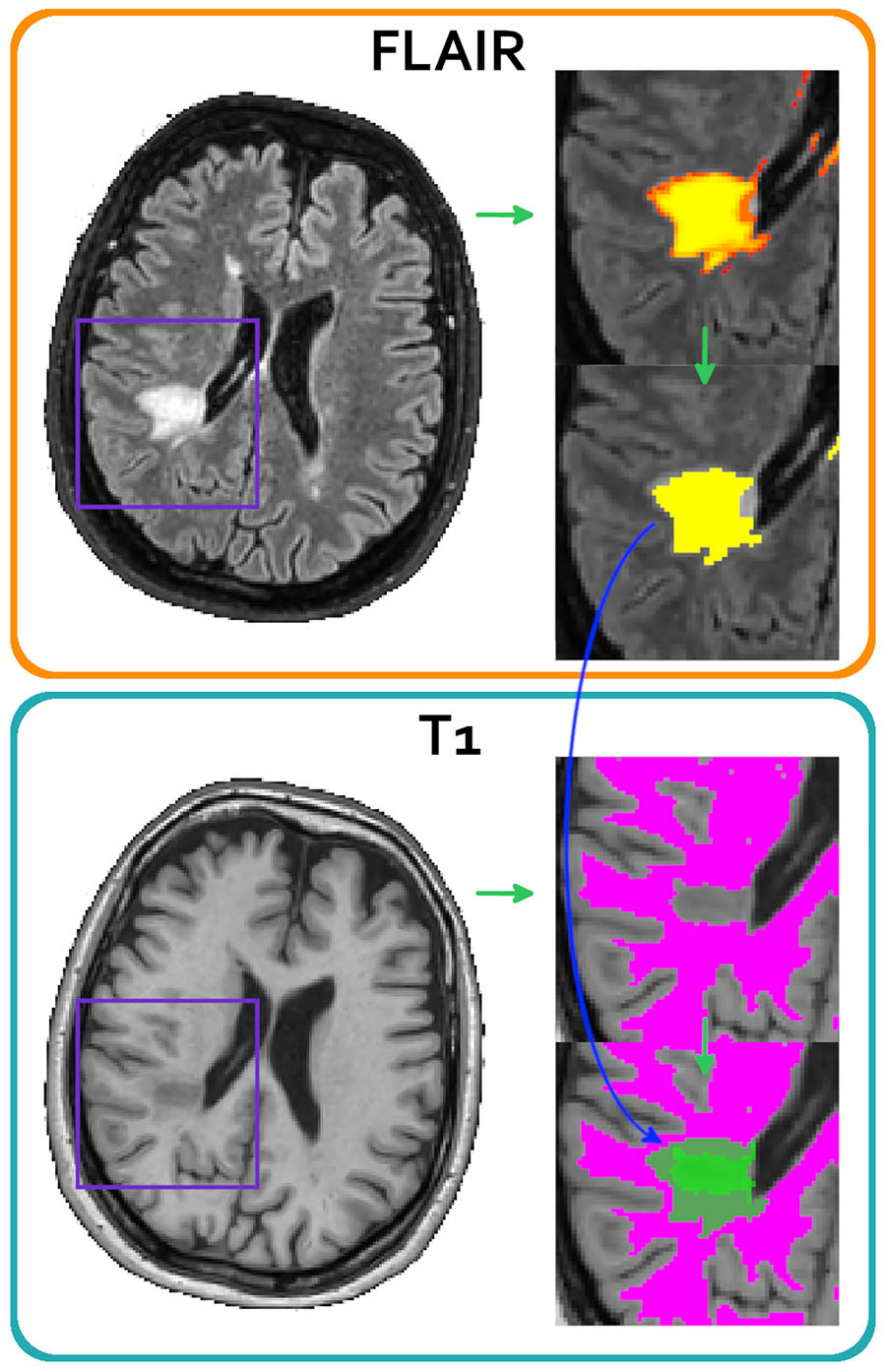
Lesion segmentation process on FLAIR and T1 images. Lesions identified with Lesion Segmentation Tool (LST) on FLAIR images (orange box) are represented with a probability map (top of the zoomed view); according to LST recommended usage, only the area characterized by a lesion probability >0.5 has been kept (yellow area, bottom of zoomed view). The cyan box shows white matter (WM) segmentation, where the lesion is not included in the non‐lesional WM mask (top of the zoomed view); at the bottom of the zoomed view, it is shown the lesion mask overlaid onto the WM mask: Hypo‐ and iso‐intense areas are visible as two shades of green.

The sub‐volume segmentation process is exemplarily described in Figure 2. The results of the correlation analysis between the segmented FL volumes and sub‐volumes and the EDSS are reported in Table 3, with the corresponding average percentage of the sub‐volumes. The FL volumes positively correlated (close to significance) with EDSS and the additional T1 segmentation, which allows the identification of FL sub‐volumes characterized by hypo‐intense gray value (which covered around 50% of the whole lesion volumes) showed a significant correlation (*p* < .05) with the EDSS. LST segmentation was computed both on source high‐resolution FLAIR images and after alignment to GraSE images with a consequent decrease in spatial resolution. However, the obtained results were comparable (Table 3).

**TABLE 3:** Correlation between focal lesion (FL) sub‐volumes and expanded disability status scale (EDSS).

Resolution	FL sub‐volumes	*r*‐Coefficient	*p*‐Value	% Volume
**High**	**LST**	.44	.06	100
	**T1‐iso**	.39	.09	47.8
	**T1‐hypo**	**.47**	**.04**	52.2
**Low**	**LST**	.41	.07	100
	**T1‐iso**	.42	.06	48.5
	**T1‐hypo**	**.47**	**.04**	51.5

*Note*: High‐resolution = original FLAIR images; low‐resolution = FLAIR after alignment to GraSE images.

Abbreviations: GraSE, gradient and spin echo; LST, Lesion Segmentation Tool.

**FIGURE 3:**
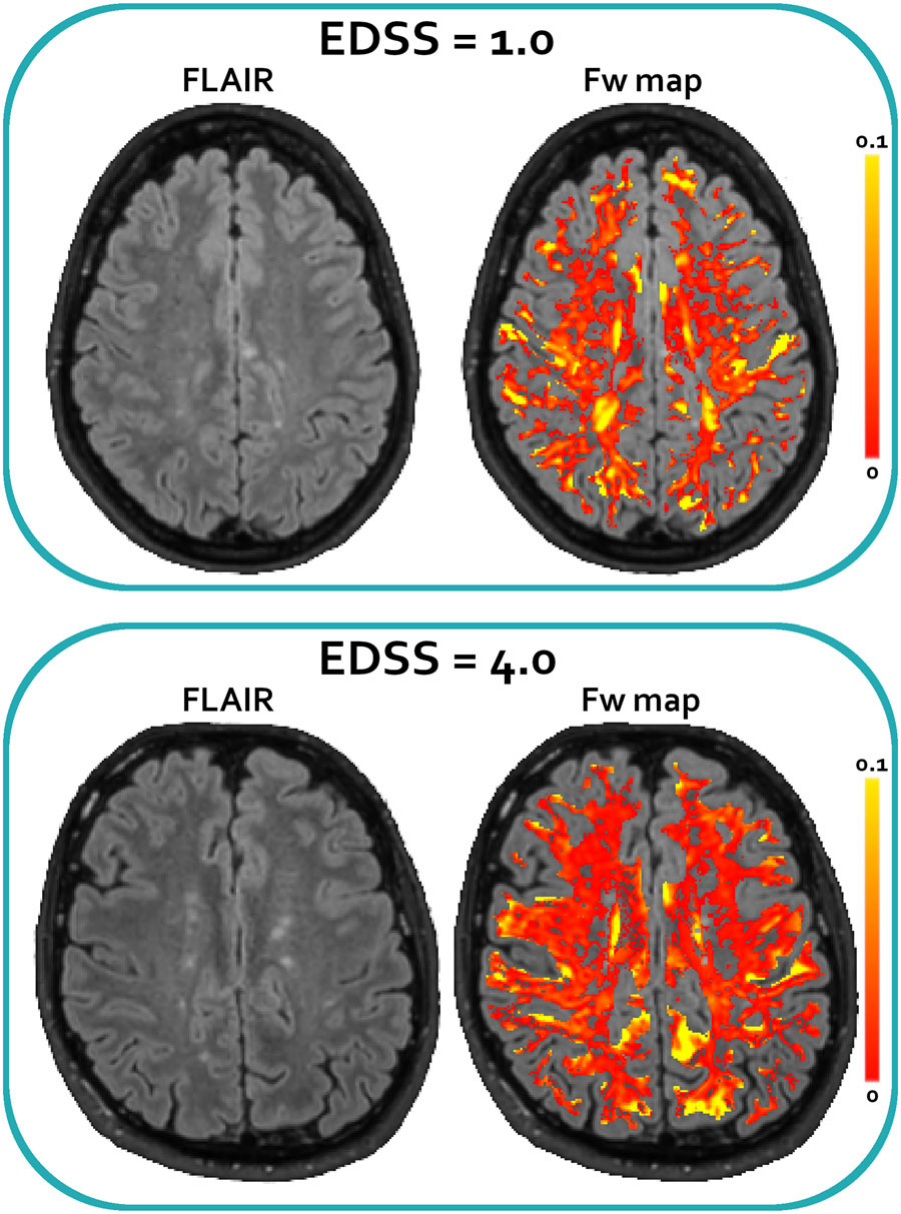
White matter T2 relaxation. Representative FLAIR (left) and free water (Fw) maps (right, FLAIR with the overlay of Fw maps) of two patients (#17 up, and #4 bottom) with expanded disability status scale (EDSS) of 1.0 and 4.0, respectively. Fw is shown as relative abundance (the sum of myelin water [Mw], intra‐extracellular water [IEw], and Fw were normalized to 1 voxel‐by‐voxel). The maps have been masked according to the eroded non‐lesional white matter (WM) mask and to the lesion areas identified with Lesion Segmentation Tool (LST). It is possible to appreciate that the patient with the higher EDSS shows a more populated Fw map.

**FIGURE 4:**
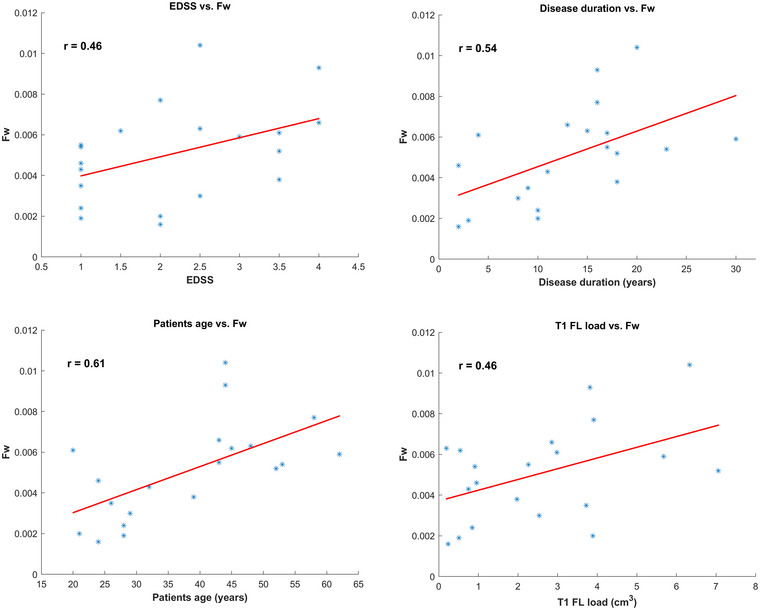
Exemplary plots, showing the correlations between median free water (Fw) and expanded disability status scale (EDSS), disease duration, age, and T1 lesion load.

The results of the correlation analysis of the quantitative T2‐relaxation parameters computed on the non‐lesional WM (*r*‐coefficients are reported in Table 4) showed that the median of Fw positively and significantly correlated (*p* < .05) with EDSS, and both the mean and the median Fw positively and significantly correlated with disease duration, age, and T1 lesion load. Exemplary Fw images of patients with different EDSS are shown in Figure 3. Exemplary plots are reported in Figure 4, showing the correlations observed between median Fw and EDSS, disease duration, age, and T1 FL load. The bivariate correlations, including both FL volumetric and WM T2‐relaxation measures, significantly correlated with EDSS (data not shown), but they never improved the significance obtained by individual volumetric measures. Accordingly, the mutual correlation analysis between T2‐relaxation parameters computed on the non‐lesional WM and the T1 hypo‐intense FL volumes (Table 4) showed that the Fw parameter that correlated with EDSS also correlated with FL volumes.

**TABLE 4:** Correlation between white matter (WM) T2‐relaxation and clinical/radiological parameters.

Metric	WM parameter	EDSS (Spearman)	Disease duration (Pearson)	Age (Pearson)	T1 FL load (Pearson)
**Median**	**Mw**	−0.01	0.08	0.18	−0.01
	**IEw**	−0.15	−0.27	−0.36	−0.15
	**Fw**	**0**.46*	**0**.54*	**0**.61*	**0**.46*
	**IEw T2**	−0.01	−0.26	−0.41	0.20
**Mean**	**Mw**	−0.02	0.09	0.20	−0.01
	**IEw**	−0.06	−0.28	−0.39	−0.15
	**Fw**	0.33	**0**.58*	**0**.54*	**0**.50*
	**IEw T2**	−0.03	−0.26	−0.40	0.18

*Note*: Significant correlation (*p < .05) are reported in bold.

Abbreviations: EDSS, expanded disability status scale; FL, focal lesions; Fw, free water; IEw, intra‐extracellular water; Mw, myelin water.

## 4 DISCUSSION

The present study aims to investigate the quantitative T2‐relaxation parameters computed on the non‐lesional WM, and their correlation with clinical disability, assessed by EDSS, disease duration, patient age, and lesion load.

In agreement with the current literature, the correlation between FL volumes and EDSS was weak, and the T1 hypo‐intense regions allowed for identifying FL sub‐volumes with a significant correlation. Interest in lesions that appear hypo‐intense on T1‐weighted images has grown because that provides more specificity for axonal loss and a closer link to neurologic disability (Valcarcel et al., 2018). Accordingly, the T1 lesion load showed the most significant MRI correlation with cognitive impairment in MS patients (Kimiskidis et al., 2016), and meta‐analysis revealed a correlation between T1 hypo‐intense lesions’ mean volume and EDSS score, with a high certainty of the evidence (Valizadeh et al., 2021).

Differently from other published studies, we did not observe a significant Mw correlation with EDSS in the non‐lesional WM. It has been reported that in MS subjects, Mw decreased over 5 years in normal‐appearing WM (Vavasour et al., 2018), and that deficient Mw volume fraction in normal‐appearing WM correlated significantly with the EDSS score (Kitzler et al., 2012). Moreover, a strong association between a pattern of Mw values in the normal‐appearing WM and cognitive performance was found, separate from the influence of FL (Baumeister et al., 2019).

Indeed, our data evidenced that in the non‐lesional WM, the median Fw significantly correlated with EDSS, and both the median and the mean Fw significantly correlated with disease duration, age, and FL volumes. That was somehow in agreement with another study, where multicomponent T2 mapping showed that the mixed water pools with a T2 above 110 ms were not related to age but strongly increased with EDSS (Baranovicova et al., 2016). As a potential source of the long‐T2 signal is an increase in extracellular water, extending the data acquisition window of the multi‐echo T2 relaxation sequence could be useful to better characterize the T2 decay in MS (Laule, Vavasour, Mädler, et al., 2007; Laule, Vavasour, Kolind, et al., 2007). Particularly, such an increase in the Fw component is suggestive of a prodromal phenomenon in brain degeneration. A recent study (Zhou et al., 2023) demonstrated that parenchymal Fw, a measure of sub‐voxel cerebrospinal fluidlike water in the brain tissue, is linearly associated with age in the WM. That study applied multi‐compartment T2 relaxometry in cognitively normal subjects. However, the main limitation of our study is the small number of patients investigated, which determines its exploratory nature rather than confirmatory, and further data are required to clarify differences between normal individuals and MS patients.

The median Fw was the only T2‐relaxation parameter that correlates with EDSS, which also correlated with FL volumes. The bivariate correlations, including both FL volumes and WM T2‐relaxation parameters, did not improve the significance of individual volumetric measures. Composite scores, including relaxation times of different tissues and/or volumetric measures, could generally correlate more strongly with EDSS than individual measures (Poonawalla et al., 2010). However, in another study, combining different WM metrics did not yield a measure more sensitive to damage than any single measure, suggesting that the metrics are at least partially correlated with each other but sensitive to different aspects of the pathology (Lipp et al., 2019).

Finally, it is worth noting that the presented analysis was fully automated, as it is advisable to monitor MS patients by quantitative MRI in repeated follow‐up examinations and longitudinal studies, providing automated clinical decision support integrated into the radiological‐routine flow (Todea et al., 2023).

## 5 CONCLUSION

In summary, in this study, stable RR MS patients were investigated by conventional imaging, WM T2 relaxation, and a fully automated post‐processing. The correlation between FL volumes and EDSS was moderate and became stronger considering T1 hypo‐intense sub‐volumes, consistent with more severe tissue damage. T2 relaxation allowed identifying subtle alterations in the non‐lesional WM, particularly an increase of the Fw component, which correlates with EDSS, disease duration, and patient age, but in these stable patients do not seem to be independent EDSS‐predictors from FL volumes. However, such an increase in the Fw component of the non‐lesional WM is suggestive of an uninvestigated prodromal phenomenon in brain degeneration, which deserves to be further investigated in future studies.

## AUTHOR CONTRIBUTIONS

**Pietro Bontempi**: Data curation; formal analysis; investigation; methodology; software; writing—original draft; writing—review and editing. **Umberto Rozzanigo**: Conceptualization; data curation; validation; writing—original draft; writing—review and editing. **Sabrina Marangoni**: Data curation; formal analysis; investigation. **Elena Fogazzi**: Data curation; methodology; software; writing—review and editing. **Daniele Ravanelli**: Investigation; methodology; software. **Lucia Cazzoletti**: Data curation; methodology; software; writing—review and editing. **Bruno Giometto**: Supervision; validation. **Paolo Farace**: Conceptualization; data curation; formal analysis; investigation; methodology; project administration; writing—original draft; writing—review and editing.

## CONFLICT OF INTEREST STATEMENT

The authors have no conflicts of interest to declare.

## FUNDING INFORMATION

None

### Peer Review

The peer review history for this article is available at https://publons.com/publon/10.1002/brb3.3334.

## Data Availability

The data that support the findings of this study are available on request from the corresponding author. The data are not publicly available due to privacy or ethical restrictions.
